# {332}<113> and {112}<111> Twin Variant Activation during Cold-Rolling of a Ti-Nb-Zr-Ta-Sn-Fe Alloy

**DOI:** 10.3390/ma15196932

**Published:** 2022-10-06

**Authors:** Alexandru Dan, Elisabeta Mirela Cojocaru, Doina Raducanu, Anna Nocivin, Ion Cinca, Vasile Danut Cojocaru

**Affiliations:** 1 Department of Metallic Materials Processing, University Politehnica of Bucharest, 060042 Bucharest, Romania; 2 Department of Industrial Management, Ovidius University of Constanța, 900527 Constanța, Romania

**Keywords:** β-Ti phase, twinning, cold-deformation, microstructure

## Abstract

Deformation twinning is a phenomenon that causes local shear strain concentrations, with the material either experiencing elongation (and thus a tensile stress) or contraction (compressive stress) along the stress directions. Thus, in order to gauge the performance of the alloy better, it is imperative to predict the activation of twinning systems successfully. The present study investigates the effects of deformation by cold-rolling on the {332}<113> and {112}<111> twin variant activation in a Ti-30Nb-12Zr-5Ta-2Sn-1.25Fe (wt.%) (TNZTSF) alloy. The Ti-30Nb-12Zr-5Ta-2Sn-1.25Fe (wt.%) alloy was synthesized in a cold crucible induction levitation furnace, under an argon-controlled atmosphere, using high-purity elemental components. The TNZTSF alloy was cold-deformed by rolling, in one single step, with a total deformation degree (thickness reduction) of ε ≈ 1% (CR 1), ε ≈ 3% (CR 3), and ε ≈ 15% (CR 15). The microstructural investigations were carried out with the SEM-EBSD technique in order to determine the grain morphology, grain-size distribution, crystallographic orientation, accumulated strain-stress fields and Schmid Factor (SF) analysis, all necessary to identify the active twin variants. The EBSD data were processed using an MTEX Toolbox ver. 5.7.0 software package. The results indicated that the TNZTSF alloy’s initial microstructure consists of a homogeneous β-Ti single phase that exhibits equiaxed polyhedral grains and an average grain-size close to 71 μm. It was shown that even starting with a 1% total deformation degree, the microstructure shows the presence of the {332}<113> twinning ((233)[$\bar{3}11$] active twin variant; Schmit factor SF = −0.487); at a 3% total deformation degree, one can notice the presence of primary and secondary twin variants within the same grain belonging to the same {332}<113> twinning system (($32\bar{3}$)[$1\bar{3}\bar{1}$] primary twin variant—SF = −0.460; ($23\bar{3}$)[$\bar{3}1\bar{1}$] secondary twin variant—SF = −0.451), while, at a 15% total deformation degree, besides the {332}<113> twinning system, one can notice the activation of the {112}<111> twinning system (($1\bar{1}2$)[$\bar{1}11$] active twin variant—SF = −0.440). This study shows the {332}<113> and {112}<111> twinning variant activation during cold-deformation by rolling in the case of a Ti-30Nb-12Zr-5Ta-2Sn-1.25Fe (wt.%) (TNZTSF) alloy.

## 1. Introduction

Titanium alloys are materials that possess a high number of desirable characteristics, including having a lower density than that of iron while maintaining a tensile strength similar to that of most steels, as well as possessing a high resistance to corrosive mediums and a high tensile strength. Moreover, they have a high melting temperature, as well as good thermal transfer properties and can be easily cast and/or forged [1]. It is thus easy to understand why they see numerous uses in a wide range of fields, with examples including the automotive and aerospace industries (engine components), chemical and nuclear applications (reservoirs, thermal transfer equipment), or the biomedical field (implantable devices) [2,3].

In the past decade, a lot of effort was put into the study of multicomponent Ti alloys that were created with alloying elements that are both non-toxic and non-allergic (for example, Ta, Nb, Zr, Sn, and Fe), as they offer a meld of good mechanical properties, a high resistance to corrosive environments/fluids, as well as an enhanced biocompatibility [4,5,6]. When it comes to the mechanical properties, such as the requirement for a low elastic modulus in implants, it was demonstrated that the values of these parameters are highly influenced by the thermomechanical processing conditions [7,8]. As such, having an adequate thermomechanical processing route is of utmost importance when it comes to obtaining microstructures with a favorable phase combination that would yield the desired mix of properties.

During the last decade, two distinctive directions were followed in the development of multicomponent Ti alloys, either controlling the properties by the refinement of α precipitation [3,4] and/or by promoting twinning induced plasticity (TWIP)/transformation induced plasticity (TRIP) [5,6,7]. However, with thermomechanical processing and, implicitly, cold-rolling, comes the propensity for deformation twining. Indeed, it was observed that the formation of twins is closely correlated with a high strain-hardening rate [9]. Generally, twinning can be defined with the help of a unit sphere and the pair {***K***}<***η***>, where ***K*** represents the twinning plane which intersects the sphere on ***η***, the twinning/shear direction [10]. In the case of most stable bcc metals, the twinning system that is usually observed is {112}<111> [11]. However, it was reported that β-Ti alloys exhibit twinning belonging to the {332}<113> system [11,12,13,14]. It was suggested in recent studies that the {332}<113> system plays an important role in the work hardening of β-Ti alloys, and thus that it is a factor in improving their mechanical properties [15]. Deformation twinning is a phenomenon that causes local shear strain concentrations, with the material either experiencing elongation (and thus a tensile stress) or contraction (compressive stress) along the stress directions [16]. Thus, in order to gauge the performance of the alloy better, it is imperative to predict the activation of twinning systems successfully.

This paper provides studies on the effect of the thermomechanical processing on the deformation twinning in a Ti-30Nb-12Zr-5Ta-2Sn-1.25Fe (wt.%) (TNZTSF) alloy by subjecting it to cold rolling at different total deformation degrees: ε ≈ 1% (CR 1), ε ≈ 3% (CR 3), and ε ≈ 15% (CR 15). The plastically deformed samples, as well as the initial (I) state, are subjected to several types of investigation: chemical composition (EDS), grain morphology, grain-size distribution, CSL (Coincident Site Lattice), crystallographic orientation (IPF map), accumulated strain-stress fields (GROD, KAM), a Schmid Factor (SF) analysis being performed in order to identify the active twinning variant.

## 2. Materials and Methods

### 2.1. Alloy Synthesis

The Ti-30Nb-12Zr-5Ta-2Sn-1.25Fe (wt.%) (TNZTSF) alloy was synthesized in an inert controlled atmosphere (argon) with the aid of a FIVE CELES-MP25 (Five’s Group Company, Paris, France) cold crucible induction levitation furnace, using only high-purity elements: Ti: min. 99.6%, no. GF71176776; Nb: min. 99.9%, no. GF49338120; Zr: min. 99.5%, no. GF10742284; Ta: min. 99.9%, no. GF80066392; Sn: min. 99.96%, no. GF11140928; and Fe: min. 99.98%, no. 267945 (SIGMA ALDRICH/MERCH, Merck KGaA, Darmstadt, Germany). In order to ensure that the alloy possessed a high chemical homogeneity, the obtained ingots were re-melted three times. The chemical composition of the alloy was determined by the EDS technique, using a TESCAN VEGA II—XMU (TESCAN, Brno, Czech Republic) scanning electron microscope (SEM), coupled with a BRUKER Quantax xFlash 6/30 EDS (Bruker Corporation, Billerica, MA, USA) detector.

### 2.2. Thermomechanical Processing Route

**Figure 1:**
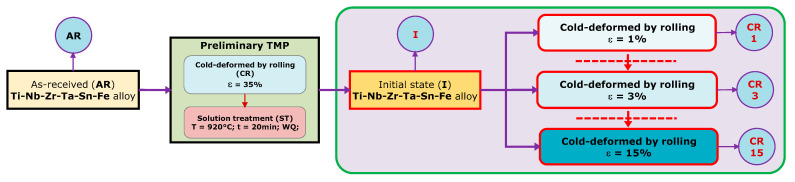
Applied thermomechanical processing (TMP) route to the TNZTSF alloy.

Figure 1 showcases the utilized thermomechanical processing route (TMP), beginning with the as-received (AR) TNZTSF alloy. To begin with, the alloy was cold-deformed by rolling (CR), using a Mario di Maio LQR120AS (Mario di Maio Inc., Milano, Italy) rolling-mill, with a total deformation degree (reduction in thickness) of approx. ε ≈ 35%, in three equal steps. After CR, as a second step, the TNZTSF samples were subjected to a solution treatment at 920 °C (ST) for a total time of 20 min. The solution treatment was followed by water quenching (WQ). The ST treatment was realized using a CARBOLITE-GERO SR 100 × 500 (Carbolite-Gero Inc., Neuhausen, Germany) furnace in high-vacuum conditions. The purpose of the preliminary TMP was to induce the formation of a pristine microstructure, consisting of a homogeneous equiaxed **β**-Ti phase, with an average grain-size in the range of 60–80 μm, and low remanent strain-stress fields. The resulted microstructural state counted as the initial (I) state for the final cold deformation, which was performed in one step, with a total deformation degree of ε ≈ 1% (CR 1), ε ≈ 3% (CR 3), and ε ≈ 15% (CR 15). The I, CR 1, CR 3, and CR 15 states were further microstructurally investigated.

### 2.3. Microstructural Characterization

**Figure 2:**
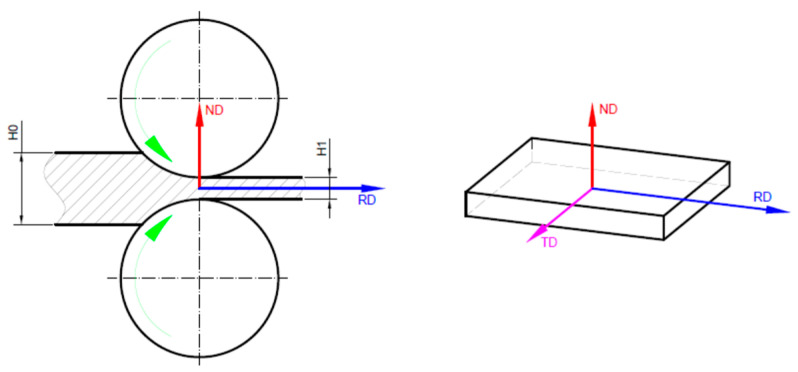
Sample reference system.

The microstructures of the I, CR 1, CR 3, and CR 15 states were analyzed in order to investigate the twinning deformation mechanism occurring during cold deformation. All microstructural investigations were carried out in the RD-TD sample reference plane (see Figure 2). A Metkon MICRACUT 202 (Metkon Instruments Inc., Bursa, Turkey) high-precision cutting equipment, that was fitted with a NX-MET XDLM (NX-MET, Echirolles, France) diamond cutting disk, was utilized in order to cut samples from all states. SAID samples were then hot-mounted in a conductive phenolic resin with the aid of a Buehler SimpliMet2 (Buehler Ltd., Lake Bluff, IL, USA) hot-mounting press. The mounted samples were first polished by a MetkonDigiprep ACCURA (Metkon Instruments Inc., Bursa, Turkey) machine. In order to improve the quality of the samples’ surfaces further, this was followed by an additional super-polishing step that was carried out by a Buehler VibroMet2 (Buehler Ltd., Lake Bluff, IL, USA) machine. The polishing and super-polishing steps are explained in greater detail in a previous paper [17].

The microstructural investigation was performed by XRD and SEM techniques. The XRD characterization was performed using a RIGAKU MiniFlex600 (RIGAKU, Tokyo, Japan) benchtop diffractometer, with the patterns, in 2θ, investigated between 30° to 90° and Cu-Kα radiation, providing a detection limit in the range of 0.1 to 1 wt.%, while the SEM characterization was carried out with a TESCAN VEGA II SEM microscope fitted with a Bruker eFlash1000 EBSD (Bruker Corporation, Billerica, MA, USA) detector. During the EBSD measurements, the following parameters were utilized: 320 × 240 pixel EBSD resolution, 512 × 512 pixel image size, 10 ms acquisition time/pixel, 1 × 1 binning size, and less than 2% zero solutions. In the EBSD analysis, the constituent β-Ti phase was indexed in the BCC (body centered cubic) system. The lattice parameter was a = 3.293 Å. No other secondary phases were detected. The EBSD data were processed using a MTEX Toolbox ver. 5.7.0 software package.

## 3. Results

### 3.1. Initial (I) TNZTSF Alloy

**Figure 3:**
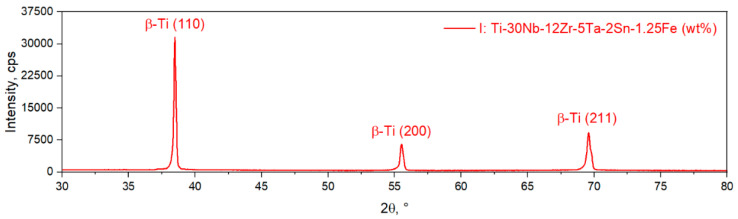
XRD spectra of the TNZTSF alloy in its initial (I) state.

A typical XRD spectra of the TNZTSF alloy in its initial (I) state is showcased in Figure 3. The X-ray diffraction spectra confirmed that the alloy’s microstructure is composed solely of the β-Ti phase with its characteristic peaks being observed at 2θ = 38.65°, 55.81°, and 69.95°, corresponding to the (110), (200), and (211) reflection planes. The β-Ti phase was indexed in the 229 BCC system. The calculated lattice parameter was a = 3.293Å. No other secondary phases were detected.

**Figure 4:**
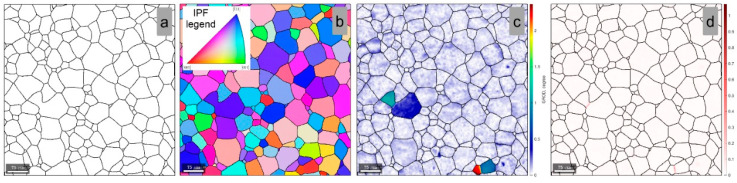
Grains boundary (**a**); IPF-Z map (**b**); GROD map (**c**); KAM map within the initial (I) TNZTSF alloy microstructure (**d**).

Figure 4a shows a microstructural image that is typical of the initial (I) state of the TNZTSF alloy and presents the grain morphology within the microstructure. The β-Ti phase grains show a polyhedral equiaxed morphology with a narrow grain-size distribution. The crystallographic orientation of the observed grains, expressed by the Inverse Pole Figure with respect to the Z sample axis (IPF-Z map), is presented in Figure 4b. One can observe that the grains tend to show an orientation spread from [001] to [$\bar{1}11$] directions, parallel to the Z sample axis (ND sample reference direction). The Grain Reference Orientation Distribution (GROD) map can be used, at the microstructural level, to assess accumulated strain-stress fields, using as the reference the average orientation of the considered grain [18,19,20,21,22,23,24,25]. When it comes to the initial state (I) of the TNZTSF alloy, the GROD distribution map (Figure 4c) shows that the β-Ti phase has low-stressed grains, with the maximal value of GROD being 2.3°. Furthermore, the β-Ti phase shows an almost uniform GROD distribution, indicating a low susceptibility to the formation of microcracks during TMP.

The Kernel Average Misorientation (KAM) analysis can be used as a tool for visualization of accumulated strain-stress fields; considering that KAM shows a measure of local grain misorientation, it can also be used to reveal local dislocation structures in the TMP-processed alloy [26,27,28,29,30,31,32,33,34,35]. The KAM distribution map of the TNZTSF alloy in its initial (I) state (Figure 4d) shows that the β-Ti phase is also presenting an almost uniform KAM distribution, with low-strained grains, with a maximum KAM of 1.1° recorded near grain boundaries.

**Figure 5:**
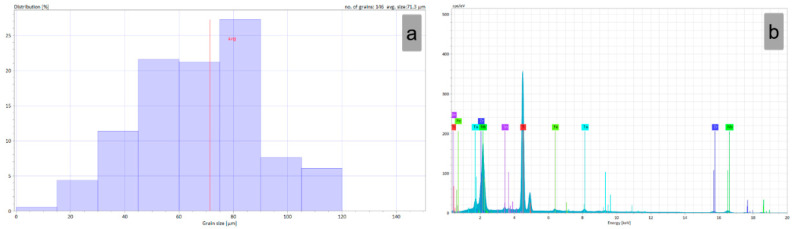
Grain-size distribution (**a**) and EDS spectra of the TNZTSF alloy in its initial (I) state (**b**).

Figure 5a showcases the grain-size distribution of the TNZTSF alloy in its initial (I) state, which revealed grains with an average size close to 71 μm, with most of the grains (>75%) within the 55 μm to 85 μm range. Figure 5b shows the EDS spectra of the initial (I) TNZTSF alloy, in which one can identify the presence of only the desired alloying elements (Ti, Nb, Zr, Ta, Sn, and Fe). The computed chemical composition is presented in Table 1. Due to limitations in the SEM-EDS technique, the presence of elements with a low atomic number (such as O, N, C) was not quantified.

### 3.2. Twinning Deformation during Cold-Deformed by Rolling (CR) in TNZTSF Alloy

The β-Ti phase can accommodate the applied plastic strain eighter by dislocation slip, twinning, or stress-induced β-Ti ➔ α”-Ti*/ω*-Ti transformation. It was found that the solute content of β-stabilizing elements can influence the dominant accommodation mechanism, demonstrating that the dislocation slip is the dominant accommodation mechanism in stable β titanium alloys, while, in metastable β titanium alloys, the twinning and/or stress-induced transformation was more predominant [15,36,37]. Moreover, it was found that both {112}<111> and {332}<113> twinning systems can be present in β titanium alloys [35,36]. In terms of activation, it was found that, in stable β titanium alloys, the accommodation of the applied plastic strain is assured by the {112}<111> twinning, while, in metastable β titanium alloys, the {332}<113> twinning was a predominant mechanism [38,39].

Furthermore, it was found that the activation of {332}<113> twining is closely related to the stress-induced β-Ti ➔ α”-Ti and/or β-Ti ➔ *ω*-Ti transformation, assuming, firstly, the occurrence of β-Ti ➔ α”-Ti*/ω*-Ti and, secondly, the reversion of α”-Ti*/ω*-Ti ➔ β-Ti and activation of {332}<113> twinning [15,40,41,42,43] due to the successive slip of <113> partial dislocations on pairs of neighboring {332} planes and a subsequent shuffle of these paired planes towards each other along the <332> directions. Such shuffling modes are possible to be induced in the metastable β titanium alloys due to its low phase stability [8,40,41,42,43].

**Figure 6:**
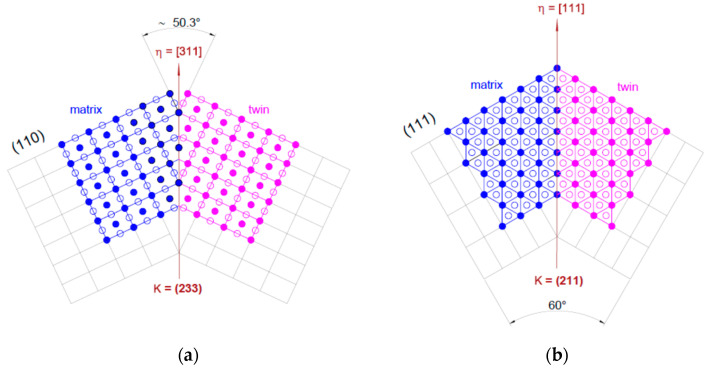
Schematic representation of the {332}<113> (**a**) and {112}<111> (**b**) twinning systems.

Generally, twinning can be defined by pair {***K***}<***η***>, where ***K*** represents the twinning plane and ***η*** the twinning/shear direction. In the case of β-Ti phase grains, the most common observed twins belong to the {332}<113> and {112}<111> twinning systems. Figure 6a shows the schematic representation of the {332}<113> twinning system, in which one can observe that, in the (110) atomic plane between the matrix and the twin, an axis rotation close to 50.3° is recorded, indicating a Σ11 CSL (Coincident Site Lattice) boundary between the matrix and the twin. In the case of the {112}<111> twinning system, in which one can observe that, in the (111) atomic plane between the matrix and the twin, an axis rotation close to 60° is recorded, indicating a Σ3 CSL boundary (see Figure 6b).

**Figure 7:**
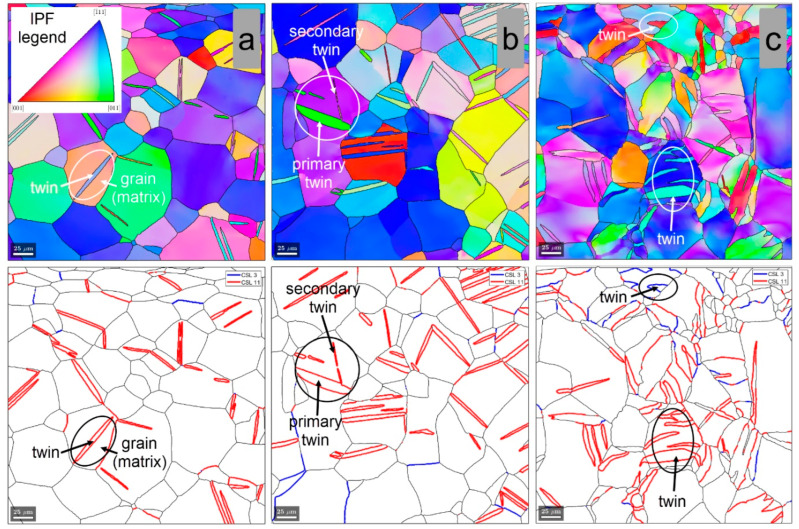
IPF-Z and grain boundary distribution within the microstructure of cold-deformed (CR) by 1% (**a**), 3% (**b**), and 15% (**c**) TNZTSF alloy.

Figure 7 shows the IPF-Z and grain boundary distribution within the microstructure of the cold-deformed (CR) TNZTSF alloy by a total deformation degree (thickness reduction) of 1% (Figure 7a), 3% (Figure 7b), and 15% (Figure 7c). As observed, the CR processing with a 1% deformation degree leads to primary twins’ development in favorably oriented β-Ti phase grains, the observed twins showing lenticular morphology with the lenticule length along the twinning direction and lenticule thickness within the twinning plane (Figure 7a). Moreover, one can observe that the developed twins are characterized by a Σ11 CSL boundary orientation relation, indicating that the developed twins belongs to the {332}<113> twinning system. When increasing the applied deformation degree to 3%, besides the primary twins, one can observe the development of twins with different orientations within the same parent grain, indicating the activation of secondary twinning variants of the same {332}<113> twinning system (Figure 7b). A different twinning system was activated starting with the CR processing at a 15% deformation degree when, besides the {332}<113> twinning system, the first signs of the {112}<111> twinning system were also observed due to the appearance of Σ3 CSL boundaries within the deformed β-Ti phase grains (Figure 7c).

In order to identify the twinning variants, a Schmid Factor (SF) analysis can be used. In the case of twinning, similarly with the case of slip, the SF can be defined as:

(1) SF=cosλcosφ

where *λ* is the angle between the stress direction and the normal of the twinning plane K, and *φ* is the angle between the stress direction and the twinning direction η. In order to obtain a SF variation between −0.5 to +0.5, the values of *λ* and *φ* are taken between 0° and 180°. According to this convention, an elongation along the stress direction will accommodate a tensile stress and, therefore, will lead to a positive SF. The activation of a variant whose SF is negative would lead to contraction along the stress direction and will accommodate a compressive stress. In titanium-based alloys, the SF analysis was successfully used to predict the activation of the twinning systems [44,45,46].

**Figure 8:**
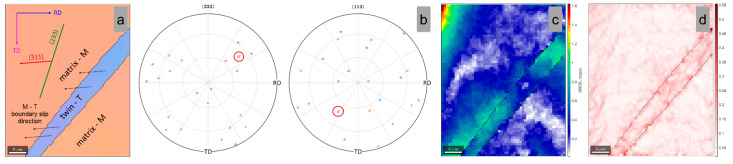
IPF-Z map (**a**); (332) and (113) Pole Figures (**b**); GROD map (**c**); KAM map (**d**) of the analyzed matrix-twin relation for the 1% cold-deformed by rolling TNZTSF alloy (CR 1).

Figure 8a shows a high-magnification image of the indicated matrix-twin relation from Figure 7a, showing the orientation of the developed {332}<113> twin. In order to confirm the {332}<113> twin, the pole figures (PF) of <332> and <113> crystal directions were analyzed (Figure 8b). One can observe that one common <332> and <113> crystallographic axis for the twinned and the matrix grain region was observed, confirming that the observed twin belongs to the {332}<113> twinning system. The GROD distribution map of the twinned and the matrix grain region (Figure 8c) shows that the twinned area is a more stressed area in comparison with the adjacent matrix (max. GROD close to 1.6°). Moreover, KAM analysis shows that the twinned area is accommodating the strain field, by local dislocation structures, induced during cold deformation (Figure 8d) (max. KAM close to 0.56°).

In order to identify the active variant {332}<113> twinning system, the SF was computed for all 12 possible variants of the {332}<113> twinning system. The computation considered the local crystallographic orientation of the matrix and the compressive stress direction parallel with the ND sample reference direction. The computed SF values are presented in Table 2. Taking into consideration that the twins are induced by a compressive stress, one must consider as possible active variants only the ones with negative SFs. In the twin variant activation analysis procedure, it was considered that only variants whose SF < −0.4 can be activated, a consideration which was confirmed for every analyzed area.

As shown in Table 2, the highest negative SF variant is represented by the (233)[$\bar{3}11$] twin variant, which shows a value close to SF = −0.487. The spatial orientation of the active (233)[$\bar{3}11$] twin variant is presented in Figure 8a, in which one can observe that the twinning [$\bar{3}11$] shear direction nicely aligns with the matrix-twin boundary slip direction.

**Figure 9:**
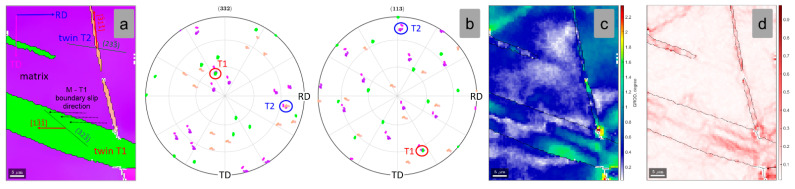
IPF-Z map (**a**); (332) and (113) Pole Figures (**b**); GROD map (**c**); KAM map (**d**) of the analyzed matrix-twin relation for the 3% cold-deformed by rolling TNZTSF alloy (CR 3).

When increasing the applied plastic strain, by applying a higher total deformation degree, one can induce the activation of primary and secondary twin variants within the same parent grain. Figure 9a shows a high-magnification image of the indicated matrix-twin relation from Figure 7b, showing the orientation of the developed {332}<113> primary and secondary twins. In order to confirm the {332}<113> twin, the pole figures (PF) of the <332> and <113> crystal directions were analyzed (Figure 9b). One can observe that one common <332> and <113> crystallographic axis was observed for both the primary twinned and the matrix region and, also, for the secondary twinned and the matrix region, confirming that both primary and secondary twins belong to the same {332}<113> twinning system. Moreover, in this case, the GROD distribution map of the twinned and the matrix region (Figure 9c) shows that the twinned area is a more stressed area in comparison with the adjacent matrix (max. GROD close to 2.2°), and the KAM analysis shows that the twins are accommodating the strain field, by local dislocation structures, induced during cold deformation (Figure 9d) (max. KAM close to 0.96°).

The computed SF values for the {332}<113> twinning system are presented in Table 3. As shown in Table 3, the highest negative SF variant (primary twin variant) is represented by the ($32\bar{3}$)[$1\bar{3}\bar{1}$] twin variant, which shows a value close to SF = −0.460, followed by the ($23\bar{3}$)[$\bar{3}1\bar{1}$] secondary twin variant, which shows a smaller value, close to SF = −0.451. Moreover, in this case, one can observe that both the primary twinning [$1\bar{3}\bar{1}$] and secondary [$\bar{3}1\bar{1}]\;$twinning shear directions are aligning with the matrix-twin boundary slip directions.

**Figure 10:**
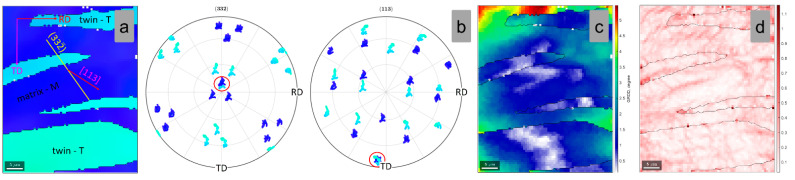
IPF-Z map (**a**); (332) and (113) Pole Figures (**b**); GROD map (**c**); KAM map (**d**) of the analyzed matrix-twin relation for the 15% cold-deformed by rolling TNZTSF alloy (CR 15).

Further increasing the applied plastic strain induces, firstly, an increase in the weight fraction of twinned regions and, secondly, the activation of different twinning systems within the same parent grain. Figure 10a shows a high-magnification image of the indicated matrix-twin relation from Figure 7c, showing the orientation of the developed {332}<113> twin. In order to confirm the {332}<113> twin, the pole figures (PF) of <332> and <113> crystal directions were analyzed (Figure 10b), in which one can observe only one common <332> and <113> crystallographic axis for the twinned and the matrix region, confirming the {332}<113> twin. Moreover, in this case, the GROD (Figure 10c) and KAM (Figure 10d) distribution maps of the twinned and the matrix region are indicating that the twinned area is a more strain-stressed area in comparison with the adjacent matrix (max. GROD close to 5.5°; max. KAM close to 1.14°). As shown in Table 4, the highest negative SF variant is represented by the ($33\bar{2}$)[113] twin variant, which shows a value close to SF = −0.458.

**Figure 11:**
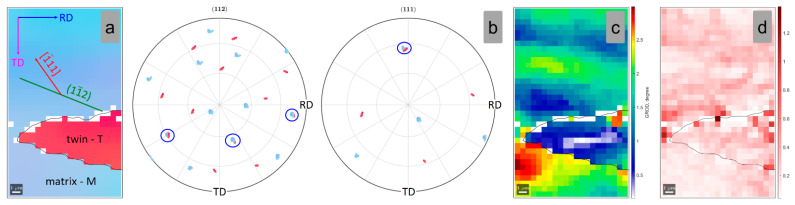
IPF-Z map (**a**); (112) and (111) Pole Figures (**b**); GROD map (**c**); KAM map (**d**) of the analyzed matrix-twin relation for the 15% cold-deformed by rolling TNZTSF alloy (CR 15).

Figure 11a shows a high-magnification image of the indicated matrix-twin relation from Figure 7c, showing the development of a different twinning system. The developed twin shows a Σ3 CSL boundary relation with the matrix β-Ti phase, indicating the {112}<111> twinning system. In order to confirm the {112}<111> twin, the pole figures (PF) of the <112> and <111> crystal directions were analyzed (Figure 11b). One can observe three common <112> and one common <111> crystallographic axes for the twinned and the matrix region, confirming the development of the {112}<111> twinning system. Moreover, in this case, the GROD (Figure 11c) and KAM (Figure 11d) distribution maps of the twinned and the matrix region are indicating that the twinned area is accommodating the increased strain-stress field (max. GROD close to 2.4°; max. KAM close to 1.28°).

In order to identify the active variant {112}<111> twinning system, the SF was computed for all 12 possible variants of the {112}<111> twinning system. Moreover, in this case, the computation considered the local crystallographic orientation of the matrix and the compressive stress direction parallel with the ND sample reference direction. The computed SF values are presented in Table 5. As shown in Table 5, the highest negative SF variant is represented by the ($1\bar{1}2$)[$\bar{1}11$] twin variant, which shows a value close to SF = −0.440.

## 4. Conclusions

In this article, the influence of the cold-deformation intensity (expressed by the total reduction in thickness/total applied deformation degree) on active twinning systems was studied under strict TMP processing conditions. The primary conclusions that are drawn are listed beneath:

1. A β-type Ti-30Nb-12Zr-5Ta-2Sn-1.25Fe (wt.%) (TNZTSF) alloy was synthesized with the aid of a cold crucible induction levitation furnace and subjected to an appropriate thermo-mechanical processing route in order to investigate active twinning systems as a function of the total applied deformation degree;
2. The initial (I) TNZTSF alloy microstructure is a homogeneous single-phase β-Ti that has equiaxed polyhedral grains, a narrow grain-size distribution, and an average grain-size of approximately 71 μm;
3. Different twinning systems can be activated during cold-deformation; the {332}<113> twining system was observed starting with a 1% total applied deformation degree, while the {112}<111> twinning system appears only for a total applied deformation degree larger than 15%, showing that the {332}<113> twinning system is the easiest to activate and the predominant twinning deformation system;
4. In the case of the {332}<113> twinning system, primary and secondary twin variants are observed, in the same parent grain, starting with an applied deformation degree of 3%.

## Figures and Tables

**Table 1:** Chemical composition of the investigated TNZTSF alloy.

Element	Atomic No.	Mass, [% wt.]	Mass, [% at.]	Abs. Error, [%]	Rel. Error, [%]
Titanium (Ti)	22	50.06	66.86	1.41	2.78
Niobium (Nb)	41	29.71	20.45	0.76	2.72
Zirconium (Zr)	40	11.87	8.32	0.31	2.83
Tantalum (Ta)	73	4.97	1.76	0.13	3.02
Tin (Sn)	50	2.08	1.12	0.10	3.96
Iron (Fe)	26	1.31	1.50	0.06	4.38
Total		100.00	100.00	-	-

**Table 2:** Computed SF values of the 12 variants belonging to the {332}<113> twinning system in the case of 1% cold-deformed by rolling TNZTSF alloy (CR 1).

{332}<113> Variants	(332) $\left[11\bar{3}\right]$	(323) $\left[1\bar{3}1\right]$	(233) $\left[\bar{3}11\right]$	$\left(3\bar{3}2\right)$ $\left[1\bar{1}\bar{3}\right]$	$\left(3\bar{2}3\right)$ [131]	$\left(2\bar{3}3\right)$ $\left[\bar{3}\bar{1}1\right]$	$\left(\bar{3}23\right)$ $\left[\bar{1}1\bar{3}\right]$	$\left(\bar{3}23\right)$ $\left[\bar{1}\bar{3}1\right]$	$\left(\bar{2}33\right)$ [311]	$\left(33\bar{2}\right)$ [113]	$\left(32\bar{3}\right)$ $\left[1\bar{3}\bar{1}\right]$	$\left(23\bar{3}\right)$ $\left[\bar{3}1\bar{1}\right]$
**SF**	−0.234	0.050	−0.487	−0.349	0.047	0.319	0.154	0.112	−0.217	−0.264	0.422	0.001

**Table 3:** Computed SF values of the 12 variants belonging to the {332}<113> twinning system in the case of 3% cold-deformed by rolling TNZTSF alloy (CR 3).

{332}<113> Variants	(332) $\left[11\bar{3}\right]$	(323) $\left[1\bar{3}1\right]$	(233) $\left[\bar{3}11\right]$	$\left(3\bar{3}2\right)$ $\left[1\bar{1}\bar{3}\right]$	$\left(3\bar{2}3\right)$ [131]	$\left(2\bar{3}3\right)$ $\left[\bar{3}\bar{1}1\right]$	$\left(\bar{3}23\right)$ $\left[\bar{1}1\bar{3}\right]$	$\left(\bar{3}23\right)$ $\left[\bar{1}\bar{3}1\right]$	$\left(\bar{2}33\right)$ [311]	$\left(33\bar{2}\right)$ [113]	$\left(32\bar{3}\right)$ $\left[1\bar{3}\bar{1}\right]$	$\left(23\bar{3}\right)$ $\left[\bar{3}1\bar{1}\right]$
**SF**	0.021	0.012	−0.127	0.044	−0.116	0.009	0.239	−0.225	0.391	0.231	−0.460	−0.451

**Table 4:** Computed SF values of the 12 variants belonging to the {332}<113> twinning system in the case of 15% cold-deformed by rolling TNZTSF alloy (CR 15).

{332}<113> Variants	(332) [$11\bar{3}$]	(323) [$1\bar{3}1$]	(233) [$\bar{3}11$]	($3\bar{3}2$) [$1\bar{1}\bar{3}$]	($3\bar{2}3$) [131]	($2\bar{3}3$) [$\bar{3}\bar{1}1$]	($\bar{3}23$) [$\bar{1}1\bar{3}$]	($\bar{3}23$) [$\bar{1}\bar{3}1$]	($\bar{2}33$) [311]	($33\bar{2}$) [113]	($32\bar{3}$) [$1\bar{3}\bar{1}$]	($23\bar{3}$) [$\bar{3}1\bar{1}$]
**SF**	0.119	−0.174	0.426	0.138	0.417	−0.190	0.123	−0.116	0.130	−0.458	−0.148	0.141

**Table 5:** Computed SF values of the 12 variants belonging to the {112}<111> twinning system in the case of 15% cold-deformed by rolling TNZTSF alloy (CR 15).

{112}<111> Variants	(112) $\left[\bar{1}\bar{1}1\right]$	(121) $\left[\bar{1}1\bar{1}\right]$	(211) $\left[1\bar{1}\bar{1}\right]$	$\left(\bar{1}12\right)$ $\left[1\bar{1}\bar{1}\right]$	$\left(\bar{1}21\right)$ $\left[11\bar{1}\right]$	$\left(\bar{2}11\right)$ $\left[\bar{1}\bar{1}\bar{1}\right]$	$\left(1\bar{1}2\right)$ $\left[\bar{1}11\right]$	$\left(1\bar{2}1\right)$ $\left[\bar{1}\bar{1}\bar{1}\right]$	$\left(2\bar{1}1\right)$ $\left[11\bar{1}\right]$	$\left(11\bar{2}\right)$ $\left[\bar{1}\bar{1}\bar{1}\right]$	$\left(12\bar{1}\right)$ $\left[\bar{1}11\right]$	$\left(21\bar{1}\right)$ $\left[1\bar{1}1\right]$
**SF**	−0.247	−0.031	−0.216	−0.295	0.085	0.235	−0.440	−0.061	0.054	0.278	0.012	−0.163

## Data Availability

The data and analysis in this study are available on request from the corresponding author.

## References

[1] Banerjee D., Williams J.C. (2013). Perspectives on titanium science and technology. Acta Mater..

[2] Gordin D.M., Ion R., Vasilescu C., Drob S.I., Cimpean A., Gloriant T. (2014). Potentiality of the “Gum Metal” titanium-based alloy for biomedical applications. Mater. Sci. Eng. C.

[3] Kolli R.P., Devaraj A. (2018). A Review of Metastable Beta Titanium Alloys. Met..

[4] Zhan L.C., Chen L.Y. (2019). A Review on Biomedical Titanium Alloys: Recent Progress and Prospect. Adv. Eng. Mater..

[5] Nnamchi P.S., Obayi C.S., Todd I., Rainforth M.W. (2015). Mechanical and Electrochemical Characterisation of new Ti-Mo-Nb-Zr Alloys for Biomedical Applications. J. Mech. Behav. Biomed. Mater..

[6] Sidhu S.S., Singh H., Gepreel M.A.H. (2021). A Review on alloy design, biological response, and strengthening of β-Titanium Alloys as biomaterials. Mater. Sci. Eng. C.

[7] Delannoy S., Baïz S., Laheurte P., Jordan L., Prima F. (2021). Elastically Graded Titanium Alloy Produced by Mechanical Surface Deformation. Front. Mater..

[8] Wang L., Lu W., Qin J., Zhang F., Zhang D. (2008). Microstructure and mechanical properties of cold-rolled TiNbTaZr biomedical β titanium alloy. Mater. Sci. Eng. A.

[9] Danard Y., Martin G., Lilensten L., Sun F., Seret A., Poulain R., Mantri S., Guillou R., Thiaudière D., von Thüngen I.F. (2021). Accommodation mechanisms in strain-transformable titanium alloys. Mater. Sci. Eng. A.

[10] Smallman R.E., Ngan A.H.W. (2014). Modern Physical Metallurgy (Eighth Edition).

[11] Gutierrez-Urrutia I., Li C.L., Ji X., Emura S., Tsuchiya K. (2018). Twinning and Detwinning Mechanisms in Beta-Ti Alloys. Mater. Sci. Forum.

[12] Zhu Y.T., Liao X.Z., Wu X.L. (2012). Deformation twinning in nanocrystalline materials. Prog. Mater. Sci..

[13] Tobe H., Kim H.Y., Inamura T., Hosoda H., Miyazaki S. (2014). Origin of {332} twinning in metastable β -Ti alloys. Acta Mater..

[14] Sun F., Zhang J.Y., Marteleur M., Gloriant T., Vermaut P., Laillé D., Castany P., Curfs C., Jacques P.J., Prima F. (2013). Investigation of early-stage deformation mechanisms in a metastable β titanium alloy showing combined twinning-induced plasticity and transformation-induced plasticity effects. Acta Mater..

[15] Lai M.J., Tasan C.C., Raabe D. (2016). On the mechanism of {332} twinning in metastable β titanium alloys. Acta Mater..

[16] Zhang J., Fu Y., Wu Y., Qian B., Chen Z., Inoue A., Wu Y., Yang Y., Sun F., Li J. (2020). Hierarchical {332}〈113〉 twinning in a metastable β Ti-alloy showing tolerance to strain localization. Mater. Res. Lett..

[17] Nocivin A., Raducanu D., Vasile B., Irimescu R., Cojocaru V.D. (2021). Tailoring a low young modulus for a beta titanium alloy by combining severe plastic deformation with solution treatment. Materials.

[18] Schayes C., Bouquerel J., Vogt J.B., Palleschi F., Zaefferer S. (2016). A comparison of EBSD based strain indicators for the study of Fe-3Si steel subjected to cyclic loading. Mater. Charact..

[19] Kamaya M. (2009). Characterization of microstructural damage due to low-cycle-fatigue by EBSD observation. Mater. Charact..

[20] Wright S.I., Nowell M.M., Field D.P. (2011). A review of strain analysis using electron backscatter diffraction. Microsc. Microanal..

[21] Kamaya M. (2012). Assessment of local deformation using EBSD: Quantification of local damage at grain boundaries. Mater. Charact..

[22] Wright S.I., Suzuki S., Nowell M.M. (2016). In situ EBSD observations of the evolution in crystallographic orientation with deformation. JOM.

[23] Littlewood P.D., Wilkinson A.J. (2012). Geometrically necessary dislocation density distributions in cyclically deformed Ti–6Al–4 V. Acta Mater..

[24] Jiang J., Britton T.B., Wilkinson A.J. (2013). Measurement of geometrically necessary dislocation density with high resolution electron backscatter diffraction: Effects of detector binning and step size. Ultramicroscopy.

[25] Jiang J., Britton T.B., Wilkinson A.J. (2013). Evolution of dislocation density distributions in copper during tensile deformation. Acta Mater..

[26] Lehto P. (2021). Adaptive domain misorientation approach for the EBSD measurement of deformation induced dislocation sub-structures. Ultramicroscopy.

[27] Rui S.-S., Han Q.-N., Wang X., Li S., Ma X., Su Y., Cai Z., Du D., Shi H.-J. (2021). Correlations between two EBSD-based metrics Kernel Average Misorientation and Image Quality on indicating dislocations of near-failure low alloy steels induced by tensile and cyclic deformations. Mater. Today Commun..

[28] Chen Y.-W., Tsai Y.-T., Tung P.-Y., Tsai S.-P., Chen C.-Y., Wang S.-H., Yang J.-R. (2018). Phase quantification in low carbon Nb-Mo bearing steel by electron backscatter diffraction technique coupled with kernel average misorientation. Mater. Charact..

[29] Kamaya M. (2009). Measurement of local plastic strain distribution of stainless steel by electron backscatter diffraction. Mater. Charact..

[30] Kamaya M. (2011). Assessment of local deformation using EBSD: Quantification of accuracy of measurement and definition of local gradient. Ultramicroscopy.

[31] Mino K., Imamura R., Koiwai H., Fukuoka C. (2003). Residual life prediction of turbine blades of aeroderivative gas turbines. Adv. Eng. Mater..

[32] Demir E., Raabe D., Zaafarani N., Zaefferer S. (2009). Investigation of the indentation size effect through the measurement of the geometrically necessary dislocations beneath small indents of different depths using EBSD tomography. Acta Mater..

[33] Field D.P., Trivedi P.B., Wright S.I., Kumar M. (2005). Analysis of local orientation gradients in deformed single crystal. Ultramicroscopy.

[34] Calcagnotto M., Ponge D., Demir E., Raabe D. (2010). Orientation gradients and geometrically necessary dislocations in ultrafine grained dual-phase steels studied by 2D and 3D EBSD. Mater. Sci. Eng. A.

[35] Wilkinson A.J., Randman D. (2010). Determination of elastic strain fields and geometrically necessary dislocation distributions near nanoindents using electron back scatter diffraction. Philos. Mag..

[36] Lai M.J., Tasan C.C., Raabe D. (2015). Deformation mechanism of ω-enriched Ti-Nb-based gum metal: Dislocation channeling and deformation induced ω-β transformation. Acta Mater..

[37] Ahmed M., Wexler D., Casillas G., Ivasishin O.M., Pereloma E.V. (2015). The influence of β phase stability on deformation mode and compressive mechanical properties of Ti-10V-3Fe-3Al alloy. Acta Mater..

[38] Xiao J., Nie Z., Tan C., Zhou G., Chen R., Li M., Yu X., Zhao X., Hui S., Ye W. (2019). Effect of reverse β-to-ω transformation on twinning and martensitic transformation in a metastable β titanium alloy. Mater. Sci. Eng. A.

[39] Xiao J.F., Nie Z.H., Ma Z.W., Liu G.F., Hao F., Tan C.W. (2020). ω precipitation: Deformation regulator in metastable titanium alloys. Mater. Sci. Eng. A.

[40] Castany P., Yang Y., Bertrand E., Gloriant T. (2016). Reversion of a parent {130}〈310〉 alpha Martensitic Twinning System at the Origin of {332}〈113〉 twins observed in metastable beta titanium alloys. Phys. Rev. Lett..

[41] Bertrand E., Castany P., Yang Y., Menou E., Gloriant T. (2016). Deformation twinning in the full-α″ martensitic Ti–25Ta–20Nb shape memory alloy. Acta Mater..

[42] Marteleur M., Sun F., Gloriant T., Vermaut P., Jacques P.J., Prima F. (2012). On the design of new b-metastable titanium alloys with improved work hardening rate thanks to simultaneous TRIP and TWIP effects. Scr. Mater..

[43] Xiao J.F., He B.B., Tan C.W. (2022). Effect of martensite on {332} twinning formation in a metastable beta titanium alloy. J. Alloys Compd..

[44] Bertrand E., Castany P., Peron I., Gloriant T. (2011). Twinning system selection in a metastable β-titanium alloy by Schmid factor analysis. Scr. Mater..

[45] Sawai T., Hishinuma A. (2005). Twin intersection in tensile deformed γ-TiAl intermetallic compounds. J. Phys. Chem. Solids.

[46] Min X., Tsuzaki K., Emura S., Sawaguchi T., Ii S., Tsuchiya K. (2013). {332}〈113〉 Twinning system selection in a β-type Ti-15Mo-5Zr polycrystalline alloy. Mater. Sci. Eng. A.

